# Growth Inhibitory Effects of *Adhatoda vasica* and Its Potential at Reducing *Listeria monocytogenes* in Chicken Meat

**DOI:** 10.3389/fmicb.2017.01260

**Published:** 2017-07-17

**Authors:** Shruti Shukla, Laxmi Ahirwal, Vivek K. Bajpai, Yun Suk Huh, Young-Kyu Han

**Affiliations:** ^1^Department of Energy and Materials Engineering, Dongguk University Seoul Seoul, South Korea; ^2^Laboratory of Plant Pathology and Microbiology, Department of Botany, Dr. Hari Singh Gour University Sagar, India; ^3^Department of Applied Microbiology and Biotechnology, Yeungnam University Gyeongsan-si, South Korea; ^4^Department of Biological Engineering, Inha University Incheon, South Korea

**Keywords:** *L. monocytogenes* NCIM 24563, *Adhatoda vasica*, ethanolic extract, antimicrobial, food model

## Abstract

The inhibitory effects of *Adhatoda vasica* ethanolic leaf extract (AVELE) against *Listeria monocytogenes* were examined to assess its potential to preserve minimally processed meat products safely. The total phenolic, flavonoid, and alkaloid levels in AVELE were 10.09 ± 4.52 mg of gallic acid equivalents (GAE)/g, 22.43 ± 1.62 mg of quercetin equivalents/g, and 19.43 ± 3.90 mg/g, respectively. AVELE (1, 5, 10, or 20%) had considerable antibacterial effects against *L. monocytogenes* NCIM 24563 in terms of the inhibitory zones (7.4–13.6 mm), MIC (100 mg/mL or 10% formulated solution), reduced cell viability, potassium ion efflux, and the release of 260-nm absorbing materials and extracellular ATP. AVELE was used as a rinse solution (5, 10, and 20%) for raw chicken breast meat. A 20% rinsing solution applied for 60 min inhibited the *L. monocytogenes* NCIM 24563 counts significantly on raw chicken breast meat. Moreover, *L. monocytogenes* NCIM 24563 did not grow in the meat sample when the rinse time was increased to 90 min at the same concentration. *L. monocytogenes* showed a greater reduction to ~3 CFU/g after rinsing with a 10 and 20% AVELE solution for 30 min than with a 5% AVELE solution. The rinsing processes with AVELE produced the final cooked chicken products with higher sensory attribute scores, such as taste, juiciness, and tenderness, compared to the control group along with a decrease in microbial contamination. Chicken meat rinsed with AVELE (rinsing time of 90 min) showed better sensory attribute scores of juiciness and tenderness, as well as the overall sensory quality compared to the untreated group. This research highlights the effectiveness of AVELE against *L. monocytogenes* NCIM 24563, suggesting that AVELE can be used as an effective antimicrobial marinade and/or a rinse for meat preservation.

## Introduction

Despite the increased understanding of the microbiology and control of infectious diseases, epidemics caused by hazardous microorganisms and the emergence of unknown disease-causing microbes pose significant public health concerns (Dussault et al., 2016). Contaminated foods are also an issue because of the serious illnesses they cause. In the United States, it has been estimated that 76 million people annually fall victim to foodborne diseases, which result in five thousand deaths per year (Dussault et al., 2016).

*Listeria monocytogenes* is a gram-positive, facultative, anaerobic bacterium, and the causative agent of listeriosis. Healthy adult humans infected with *L. monocytogenes* often remain asymptomatic (Takahashi et al., 2015), but the mortality rate in these cases ranges from 20 to 30% (McLauchlin et al., 2004). In Western countries, *L. monocytogenes* is responsible for many cases of food poisoning through dairy products, processed meats, salads, and other ready-to-eat foods that do not require heating or cooking prior to consumption (Goulet et al., 2008). Recent foodborne listeriosis outbreaks (Schlech et al., 1983; CDC, 2014) have prompted research into innovative ways of inhibiting hazardous pathogens, particularly in semi-cooked packaged meat-based food products (Aureli et al., 2000). One such method involves the use of antimicrobials to provide an increased margin of food safety and quality (Appendini and Hotchkiss, 2002).

Marination or rinsing with various seasonings or antimicrobials often reduce the growth of hazardous microbial pathogens (Perko-Mäkelä et al., 2000). The United States Department of Agriculture (USDA) recommended that individuals at risk of contracting listeriosis should avoid eating store-made or other meat-based packaged semi-cooked food products; links were established with listeriosis in a variety of semi-cooked food products (USFDA, 2011). Besides the aim of microbial reduction, additional criteria, such as consumer acceptability, human health issues, and environmental safety need to be considered before any antimicrobial agent can be implemented in food products. To ensure food-related safety, consumers are demanding natural alternatives to chemical additives in food products, but with increased food safety, and shelf-life (Piskernik et al., 2011).

Chemical preservatives are commonly added to foods to enhance food safety, but consumers are becoming increasingly concerned about the harmful effects of chemical preservatives (Marta et al., 2012). Accordingly, there is growing interest in the use of natural and renewable antimicrobials in food preservation. Moreover, plant extracts, specifically those extracted using 70% ethanol, exhibit no toxicity; hence, they could be considered safe for food applications (Ahmad and Tabassum, 2013; Mugisha et al., 2014). Plants offer a rich variety of bioactive compounds, such as phenolics, flavonoids, terpenoids, and alkaloids, with a diverse range of pharmacological activities (Bakkali et al., 2008). For example, plant extracts from rosemary, oregano, clove, thyme, and citrus fruit (lemon, orange, and grapefruit) are among the most studied natural antimicrobials for food applications (Corbo et al., 2008).

*Adhatoda vasica* (Malabar nut) is a perennial plant that is well-known in Ayurveda for its medicinal properties (Maurya and Singh, 2010; Kaur et al., 2012). The plant grows throughout India, even at higher altitudes in the Himalayan region, and is also found in Myanmar, Sri Lanka, Burma, and Malaysia. *A. vasica* has been used by indigenous people as a medicine for thousands of years, particularly in the treatment of respiratory disorders (coughs, colds, asthma, and bronchitis) (Kaur et al., 2012). *A. vasica* has been reported to possess several biological activities, which include anti-inflammatory, anti-spasmodic, anti-bleeding, anti-diabetic, and anti-jaundice effects (Maurya and Singh, 2010). Considering the potential use of natural antimicrobial agents and their application in the current scenario of food preservation, *A. vasica* has been found to exhibit potent antimicrobial activity against various foodborne pathogenic bacteria along with its *in vivo* efficacy as a food preservative to control the proliferation of *L. monocytogenes* in processed food (Subramaniam et al., 2015).

Antimicrobial coatings or washings might reduce the incidence of foodborne illnesses caused by *L. monocytogenes* in meat products. Therefore, this study examined the *in vitro* and *in vivo* efficacy of AVELE derived from *A. vasica* on the microbial load of *L. monocytogenes* NCIM 24563 in chicken meat as well as its overall efficacy as a rinse on the sensory quality of chicken meat. This is the first report to address the use of *A. vasica* in chicken to control *L. monocytogenes* NCIM 24563.

## Materials and methods

### Chemicals and reagents

Folin-Ciocalteu reagent, gallic acid, quercetin, potassium ferricyanide, ascorbic acid, and pyrogallol were purchased from Sigma Aldrich (St. Louis, MO, USA). Brain heart infusion broth (BHI broth), peptone, and brain heart infusion agar (BHI agar) were purchased from Sigma Aldrich (St. Louis, MO, USA). The spectrophotometric measurements were performed using enzyme-linked immunosorbent assay (ELISA) (Tecan, Switzerland).

### Plant materials and extract preparation

Plant material, *A. vasica* (dried leaf powder), was donated by Jeevan Herbal Products (Sagar, MP, India). Briefly, the powder (100 g) was extracted with a 20-fold volume of 70% ethanol for 3 h at 65°C, filtered through Whatman No. 2 filter paper, concentrated using a vacuum evaporator, and freeze-dried. The resulting AVELE was then stored at −20°C until further analysis.

### Analysis of total phenolic, flavonoid, and alkaloid contents of AVELE

The total phenolic contents of AVELE were determined using Folin-Ciocalteau reagent with gallic acid as the standard phenolic compound (Singleton et al., 1999). Briefly, 20 μL of the extract solution (1 mg/mL) was added to 100 mL of Folin-Ciocalteu reagent, and 80 mL of 10% aqueous sodium carbonate was added 3 min later. This mixture was left to stand for 1 h at room temperature, and the absorbance of the resulting blue colored mixture was measured at 765 nm against a 70% ethanol (200 mL) blank. The total phenolic content was calculated as the gallic acid equivalent (GAE) by the calibration curve obtained using a standard gallic acid solution. The results were expressed as mg GAE/g dry mass.

The total flavonoid content of AVELE was determined calorimetrically (Sakanaka et al., 2005). Briefly, 100 μL of AVELE or standard reagent and 400 μL of ethanol were mixed with 500 μL of a 2% AlCl_3_ solution prepared using distilled water. After 1 h incubation at room temperature, the absorbance was measured at 430 nm. Quercetin was used as a reference compound to generate a standard curve, and results are expressed as mg QE/g dry mass.

The total alkaloid content of AVELE was determined as described previously (Shamsa et al., 2008). A sample of AVELE was weighed into a 250 mL beaker, and 200 mL of 10% acetic acid in ethanol was added. The beaker was then covered and allowed to stand for 4 h at room temperature. The sample was then filtered and concentrated on a water bath to one quarter of its original volume. Concentrated ammonium hydroxide was then added dropwise to the extract until precipitation was complete. The solution was then left to stand until the precipitate had settled. The resulting precipitate was collected, washed with dilute ammonium hydroxide, and filtered. The residue (total alkaloid) was dried and weighed. The results are presented as mg/g of dried extract.

### Preparation of bacterial cultures of test pathogens

The pathogenic strain, *L. monocytogenes* NCIM 24563, which was isolated from ready-to-eat-food (serotype 1/2a), was obtained from the Microbial Type Culture Collection, Chandigarh, India. The strain was maintained on BHI agar at 4°C and grown in BHI broth at 37°C for 24 h.

Other bacterial strains, such as *Salmonella typhimurium* ATCC 43174, were obtained from the Korea Food and Drug Administration, and maintained on nutrient agar (NA) at 4°C.

### Determination of inhibitory effects of AVELE

The disc diffusion method was used to assay the anti-listerial effects of AVELE (Bajpai et al., 2014). *L. monocytogenes* NCIM 24563 was grown in BHI broth and incubated at 37°C for 18–24 h. The bacterial growth of the culture was adjusted to ~10^7^ CFU/mL, and an aliquot of the bacterial culture (100 μL) was spread over the plate count agar and dried on a clean bench for a few minutes. AVELE solutions (50 μL) at different concentrations (10, 50, 100, or 200 mg/mL, corresponding to 1, 5, 10, and 20%, respectively) were added to the discs, which were then placed on agar plates, and incubated at 37°C for 18–24 h. The zones of inhibition against *L. monocytogenes* NCIM 24563 were measured in millimeters. The assays were performed in triplicate.

### Measurement of minimum inhibitory concentration (MIC) of AVELE

A MIC microplate assay, as described by Shiu and Gibbons (2006), was used to determine the lowest AVELE concentration that inhibited the visual growth of *L. monocytogenes* NCIM 24563. Briefly, 100 μL of sterile distilled water was aliquoted into the wells of a 96-well sterile plate, and serial dilutions of an AVELE solution (200 mg/mL) were added to the cells. A standard culture (100 μL; 10^7^ CFU/mL) of *L. monocytogenes* NCIM 24563 was then dispensed into each well of a 96-well sterile plate. The plate was then sealed and incubated at 37°C for 24 h. A culture medium without the bacterial inoculum and sterilized distilled water without AVELE were used as the negative controls. To visualize the level of bacterial growth, 40 μL of *p*-iodonitrotetrazolium violet (INT) (0.04 mg/mL) per well was added and the plate was incubated at room temperature for 6 h. After incubation, the plate was examined for any color changes. The results obtained in triplicate were recorded and analyzed using Excel®.

### Effect of AVELE on viable cell counts

Tubes containing the bacterial suspension (~10^7^ CFU/mL) of *L. monocytogenes* NCIM 24563 in BHI broth (10 mL) were inoculated with AVELE at 100 mg/mL (its MIC), and maintained at 37°C (Shin et al., 2007); samples were taken at 0, 40, 80, 120, 160, and 200 min. The viable counts on BHI plates were determined using the following method. A 0.1 mL sample of each treatment was diluted 10-fold with the buffer peptone water and spread over the surface of BHI agar. The colonies were counted after incubation for 24 h at 37°C. The controls were inoculated without AVELE under the same experimental conditions. The experiments were performed in triplicate.

### Effect of AVELE on potassium ion efflux

A method devised by Bajpai et al. (2014) was used to determine the amount of potassium ion leakage. The concentration of free potassium ions in the bacterial suspensions of *L. monocytogenes* NCIM 24563 was measured after exposing the bacterial cells to AVELE at 100 mg/mL in sterile peptone water for 0, 30, 60, 90, or 120 min. The extracellular potassium concentrations were measured using a photometric procedure on a Calcium/Potassium kit (Quantofix, GmbH, Wiesbaden, Germany). The controls were devoid of AVELE. The results are expressed as the potassium concentrations (mmol/L) in the growth medium in triplicate.

### Effect of AVELE on the release of 260-nm absorbing cellular materials

This assay was conducted using 2 mL aliquots of bacterial inoculum in sterile peptone water containing 100 mg/mL of AVELE at 37°C. After exposure for 0, 30, or 60 min, the *L. monocytogenes* NCIM 24563 cells were centrifuged at 3,500 rpm, and the absorbance of the obtained supernatant was measured at 260 nm using a 96-well plate ELISA reader (Carson et al., 2002). Control sets without AVELE were also tested in an identical manner. The results are expressed in terms of the absorbance with respect to the specific time.

### Effect of AVELE on the release of extracellular adenosine 5′-triphosphate (ATP)

A previous method was adopted to determine the effects of AVELE on the membrane integrity of the tested pathogens in terms of its ability to cause the leakage of extracellular ATP (Bajpai et al., 2014). To collect the cells, actively grown cultures of *L. monocytogens* NCIM 24563 (~10^7^ CFU/mL) were centrifuged (10 min at 1,000 × g), and the collected cell pellet was washed three times with 0.1 M sodium phosphate buffer (pH 7.0) followed by centrifugation, as specified above. A 0.5 mL cell suspension (10^7^ CFU/mL) prepared in sodium phosphate buffer was placed into an Eppendorf tube for the AVELE treatment at the MIC. The samples were kept at room temperature for 30 min followed by centrifugation (5 min at 2,000 × g). To prevent the loss of ATP, the samples were incubated in ice immediately. The extracellular ATP concentrations (upper supernatant layer) were measured using an ATP bioluminescent assay kit (Sigma, MO, USA) according to the manufacturer's instructions.

### Application of AVELE in food sample

#### Determination of the pH of AVELE as a rinse solution

The pH of the prepared AVELE rinse solutions at different concentrations was determined using an In-Lab427 pH meter fitted with a combined glass electrode (Mettler-Toledo, GmbH, 8603 Schwarzenbach, Switzerland), which had been calibrated previously to pH 4.0 and 7.0.

#### Application of AVELE as a rinse in chicken meat

Raw breast chicken meat sealed-pack (250 g) was purchased from a local grocery store and stored at 4°C until analyzed. A portion (25 g) of the raw breast chicken meat was weighed and placed in a flask containing 225 mL of buffered peptone water. All the weighed samples were enriched and plated to check for the presence or absence of *L. monocytogenes* NCIM 24563 prior to artificial inoculation.

**Scheme 1:**
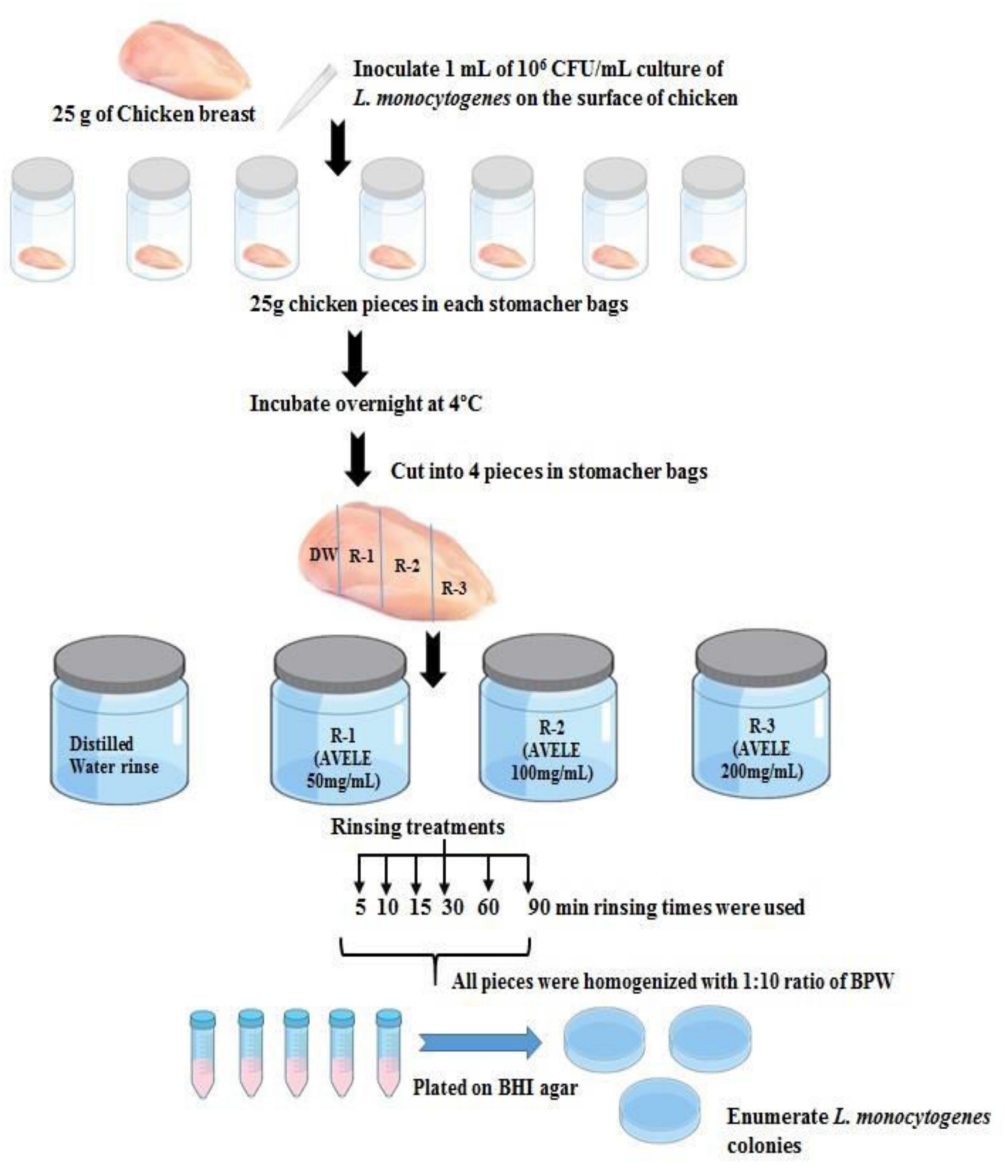
Scheme for the practical applicability of AVELE as an antimicrobial rinse for reducing L. monocytogenes NCIM 24563 in chicken meat.

The chicken breast meat samples (25 g) were then inoculated with 1 mL of an overnight culture of *L. monocytogenes* NCIM 24563 inoculum (10^6^ CFU/mL) in sterile Whirl-Pak stomacher bags (Scheme 1), which were then stored overnight at 4°C to allow bacterial attachment. The samples were then divided into four equal pieces using a sterile blade and treated with 60 mL of water (control) or AVELE autoclaved for 30 min at 121°C at concentrations of 50, 100, or 200 mg/mL. The following rinse times were used: 5, 10, 15, 30, 60, and 90 min. After rinsing, pieces of each set were removed and placed separately into sterile stomacher bags. One piece was then used to plate 0 h (no storage); the other chicken pieces were kept at 4°C overnight, held for 24 h, and plated.

To determine the *L. monocytogenes* NCIM 24563 counts in chicken meat, the samples were homogenized for 2 min at 200 rpm and diluted serially in 9 mL of buffer peptone water. Homogenized and diluted chicken samples were plated onto selective media, as mentioned above, and incubated for 24 h at 37°C. The colonies were counted, and the results are expressed as the CFU/g of the chicken meat sample. Each experiment was performed in triplicate.

Parallel sets of experiments with *L. monocytogens* NCIM 24563 and *S. typhimurium*) were also conducted using different food matrices (pork meat and ready-to eat fresh salad) to validate the industrial applications of AVELE as a rinse. Each experiment was performed in triplicate.

#### Sensory analysis of AVELE treated chicken meat samples

An evaluation of the sensory properties was conducted by an expert panel of 20 trained panelists, aged between 20 to 25 and 30 to 40 years. The panelists were selected based on their sensitivity to 4 taste attributes, odor, taste (sweet, sour, bitter, or umami), tenderness, and juiciness, to a threshold level along with a potential to confirm the taste sensory characteristics of cooked chicken (treated with the AVELE rinse solution). These sensory attributes are considered important, and a wide range of parameters are used to determine the taste properties and taste disorders of food samples. A previously reported method was followed for the descriptive sensory analysis of the test samples (Stone and Sidel, 1985). The overall sensory characteristics (odor, taste, tenderness, and juiciness) of the AVELE treated, cooked chicken samples were evaluated using a 9-point scale hedonic test principle (Stone and Sidel, 1985) and given numerical values of 1 (extremely dislike) to 9 (extremely like).

### Color analysis

The surface color of the cooked AVELE rinse-treated chicken breast samples was evaluated using a Minolta Chroma Meter CR-300 (Minolta Co. Ltd., Osaka, Japan), calibrated against a white tile. The color measurements (L^*^, a^*^, and b^*^ values representing lightness, redness, and yellowness, respectively) were taken after cooling the rinse-treated cooked samples at room temperature. For each sample, the L^*^, a^*^, and b^*^ values were taken at three locations, which included two adjacent locations to the central measurement. For color analysis, the L^*^ value indicates lightness, ranging from 0 to 100, representing dark to light (Byun et al., 2013). The a^*^ value represents the degree of red-green color, in which a higher a^*^ value indicates a redder color score. The b^*^ value represents the degree of yellow-blue color; a high positive b^*^ value indicates a yellower color score.

### Statistical analysis

All experiments were conducted in triplicate, and all data (number of colony counts) were converted to log CFUs. The means were calculated and a Duncan's test in IBM SPSS Statistics 21 was used to analyze the data. *P* values < 0.05 were considered significant.

## Results and discussion

### Total phenolic, flavonoid, and alkaloid contents of AVELE

Phenolic compounds conferring various biological and functional properties are considered to be important secondary metabolites because of their hydroxyl groups and occur ubiquitously in plants (Nithya et al., 2016). The plant materials rich in phenolics are being used increasingly in the food industry because they retard the oxidative degradation of lipids and improve the quality and nutritional value of food (Naczk and Shahidi, 2004). Flavonoids are plant-based natural components that have a range of health beneficial effects and play a significant role as scavengers or reactive oxygen species (ROS), which improve the quality of the foods (Bravo, 1998). Alkaloids are natural components of plants that have been implicated in various biological activities and used as food flavor enhancers (Koleva et al., 2012). Interestingly, in this study, AVELE contained large amounts of phenolics, flavonoids, and alkaloids with mean values of 10.09 ± 4.52 mg GAE/g, 22.43 ± 1.62 mg QE/g, and 19.43 ± 3.90 mg/g, respectively (Table 1). Vijayanandraj et al. (2014) also evaluated the phenolics and alkaloids in the *A. vasica* leaf extract and the plant was rich in alkaloids rather than phenolics. Claeson et al. (2000) reported that the plant was a rich source of quinazoline alkaloids, such as vasicine, vasicinone, deoxyvasicinone, vasicol, and adhavasicinone. Several studies have shown that plant secondary metabolites have antimicrobial activity against hazardous pathogens (Benbott et al., 2012; Maatalah et al., 2012). The biological activities of plants, including their antimicrobial activities, have been attributed to different classes of secondary metabolites, including phenolic, alkaloid, and flavonoid components (Stevenson and Hurst, 2007). In addition, these compounds contribute to the quality and nutritional value of foods by modifying their color, taste, aroma, and flavor, as well as have beneficial health effects (Iturraga et al., 2012).

**Table 1:** Total phenolic, flavonoid, and alkaloid contents in the *Adhatoda vasica* ethanolic leaf extract (AVELE).

***Adhatoda vasica*** **ethanolic leaf extract (AVELE)**		
**Total phenolic content (mg GAE)**	**Total flavonoid content (mg QE)**	**Total alkaloid (mg/g)**
10.09 ± 4.52	22.43 ± 1.62	19.43 ± 3.90

### Anti-listerial inhibitory effects

The *in vitro* inhibitory activity of AVELE against *L. monocytogenes* NCIM 24563 was assessed by measuring the diameters of the zones of inhibition. AVELE at a concentration of 20% (200 mg/mL) had an antibacterial effect against *L. monocytogenes* NCIM 24563 with an inhibitory zone diameter of 13.6 ± 1.01 mm, whereas 5 and 10% (50 and 100 mg/mL, respectively) AVELE produced the zones of inhibition of 7.4 ± 2.12 and 9.8 ± 1.54 mm, respectively (Table 2). In contrast, no inhibitory zone was observed at 1% (10 mg/mL) AVELE (Table 2). Various natural plant-based extracts have shown antimicrobial properties against various pathogens (Holley and Patel, 2005; Bakkali et al., 2008). The appropriate use of such extracts in the food industry as antimicrobials require careful selection of the undesirable organoleptic effects (Iturraga et al., 2012). Iturraga et al. (2012) reported that natural plant extracts (rosemary, oregano, and citrus) have potent antibacterial effects, as determined by the zones of inhibition against *Listeria* sp. In addition, Meignanalakshmi et al. (2013) reported that the methanolic extract (200 mg/mL) of *A. vasica* had significant antimicrobial activity against *Staphylococcus aureus, Streptococcus agalactiae, Klebsiella pneumonia, Streptococcus dysgalactiae*, and *Escherichia coli* with zones of inhibition ranging from 18.3 to 28.3 mm.

**Table 2:** Antibacterial activity of *Adhatoda vasica* ethanolic leaf extract (AVELE) against *Listeria monocytogenes* NCIM 24563.

**Concentration (AVELE)**	**Inhibitory zone (mm)**	**MIC**
10 mg/mL (1%)	–	100 mg/mL
50 mg/ mL (5%)	7.4 ± 2.12^c^	
100 mg/mL (10%)	9.8 ± 1.54^b^	
200 mg/mL (20%)	13.6 ± 1.01^a^	

*MIC, Minimum inhibitory concentration; mm, Millimeter. All values were expressed as mean ± SD of three parallel measurements (n = 3). Values in the same column with different superscripts are significantly different according to Duncan's Multiple Range Test (p < 0.05)*.

### MIC of AVELE

AVELE inhibited the growth of *L. monocytogenes* NCIM 24563 with a MIC of 10% (100 mg/mL) (Table 2), whereas 1% dimethyl sulfoxide (DMSO), as a negative control, did not inhibit its growth. In the present study, a lower MIC was obtained for AVELE, which might be due to the additive or synergistic effects of the various bioactive compounds present in AVELE. Shen et al. (2014) reported that different types of blueberry extracts had anti-listerial effects with MICs ranging from 300 to 750 mg/mL.

### Effect of AVELE on cell viability

**Figure 1:**
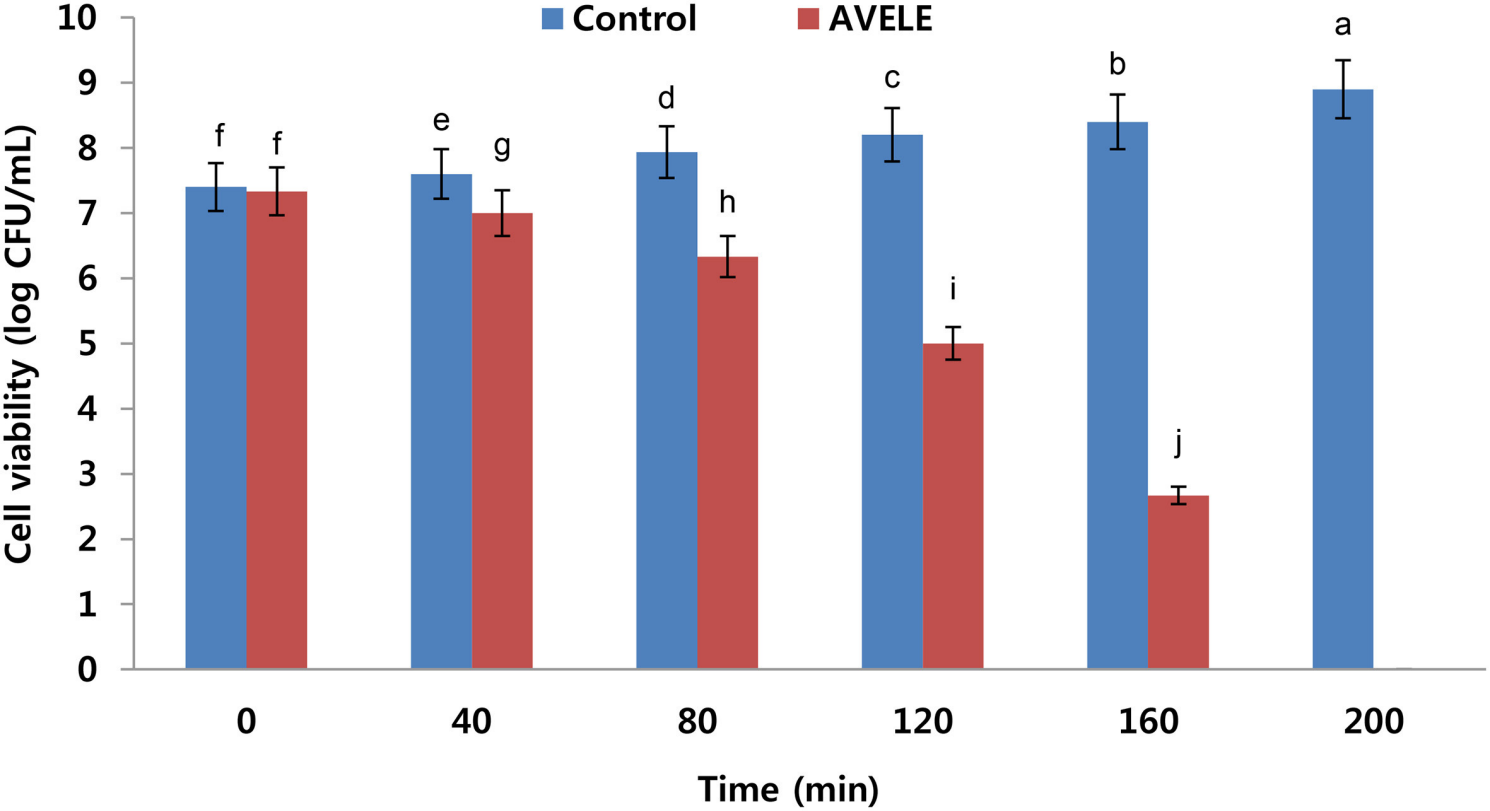
Effects of *Adhatoda vasica* ethanolic leaf extract (AVELE) on the viability of the tested foodborne pathogen, *L. monocytogenes* NCIM 24563. The data are expressed as the mean ± *SD* (*n* = 3). The values in the same column with different superscripts are significantly different according to a Duncan's multiple range test (*p* < 0.05).

AVELE inhibited the growth of *L. monocytogenes* NCIM 24563 at its MIC (Figure 1); after 40 min exposure, AVELE at its MIC strongly inhibited *L. monocytogenes*, and after 80 min, the CFU numbers declined steeply by ~4 ± 0.01 log CFU after exposure for up to 160 min. Furthermore, *L. monocytogenes* NCIM 24563 was completely eradicated after 200 min exposure (Figure 1). Higginbotham et al. (2014) reported the anti-listerial activity of the aqueous extract of *Hibiscus sabdariffa* and its use to reduce the viable counts at undetectable levels in hot dogs. They suggested that the *H. sabdariffa* extract might be a useful antimicrobial rinse for meat-based food products to control the listerial counts. Furthermore, they reported that at an extract concentration of 240 mg/mL and rinse time of 60 min, the *H. sabdariffa* extract almost eradicated *L. monocytogenes* counts in hot dogs.

### Effect of AVELE on potassium ion efflux

**Figure 2:**
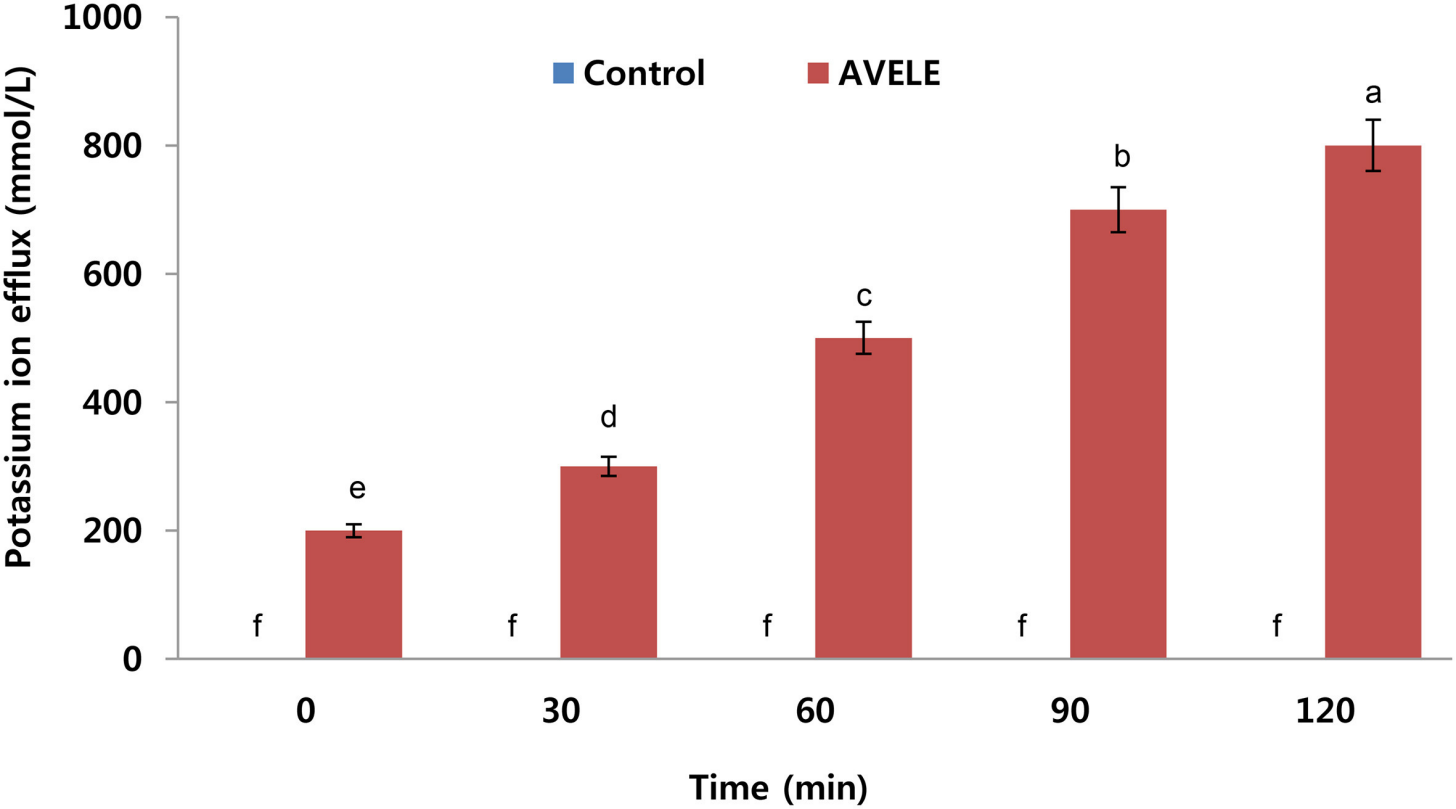
Effects of *Adhatoda vasica* ethanolic leaf extract (AVELE) on the leakage of potassium ions from the cells of *L. monocytogenes* NCIM 24563. CT refers to the control without the AVELE treatment. Data expressed as the mean ± *SD* (*n* = 3). The values in the same column with different superscripts are significantly different according to the Duncan's multiple range test (*p* < 0.05).

When *L. monocytogenes* NCIM 24563 cells were treated at its MIC (100 mg/mL), the AVELE caused the rapid release of potassium *ions* from *L. monocytogenes* NCIM 24563 cells (800 mmol/L), but no potassium leakage was observed in the control sets (Figure 2). The release of essential ions or electrolytes from the bacterial cells after treatment with an antimicrobial could be considered a good indication of its antimicrobial effect (Bajpai et al., 2014). In the present study, when tested against *L. monocytogenes* NCIM 24563, AVELE induced the release of potassium compared to the control. The bacterial plasma membrane was reported to play a pivotal role in controlling the efflux of essential ions from bacterial cells. The membrane ion permeability is closely related to the chemical composition and structures of the cell membranes. Therefore, a dramatic increase in potassium efflux from the bacterial cells indicates membrane disruption and probable cell death (Cox et al., 2001).

### Effect of AVELE on absorption at 260-nm cellular materials

**Figure 3:**
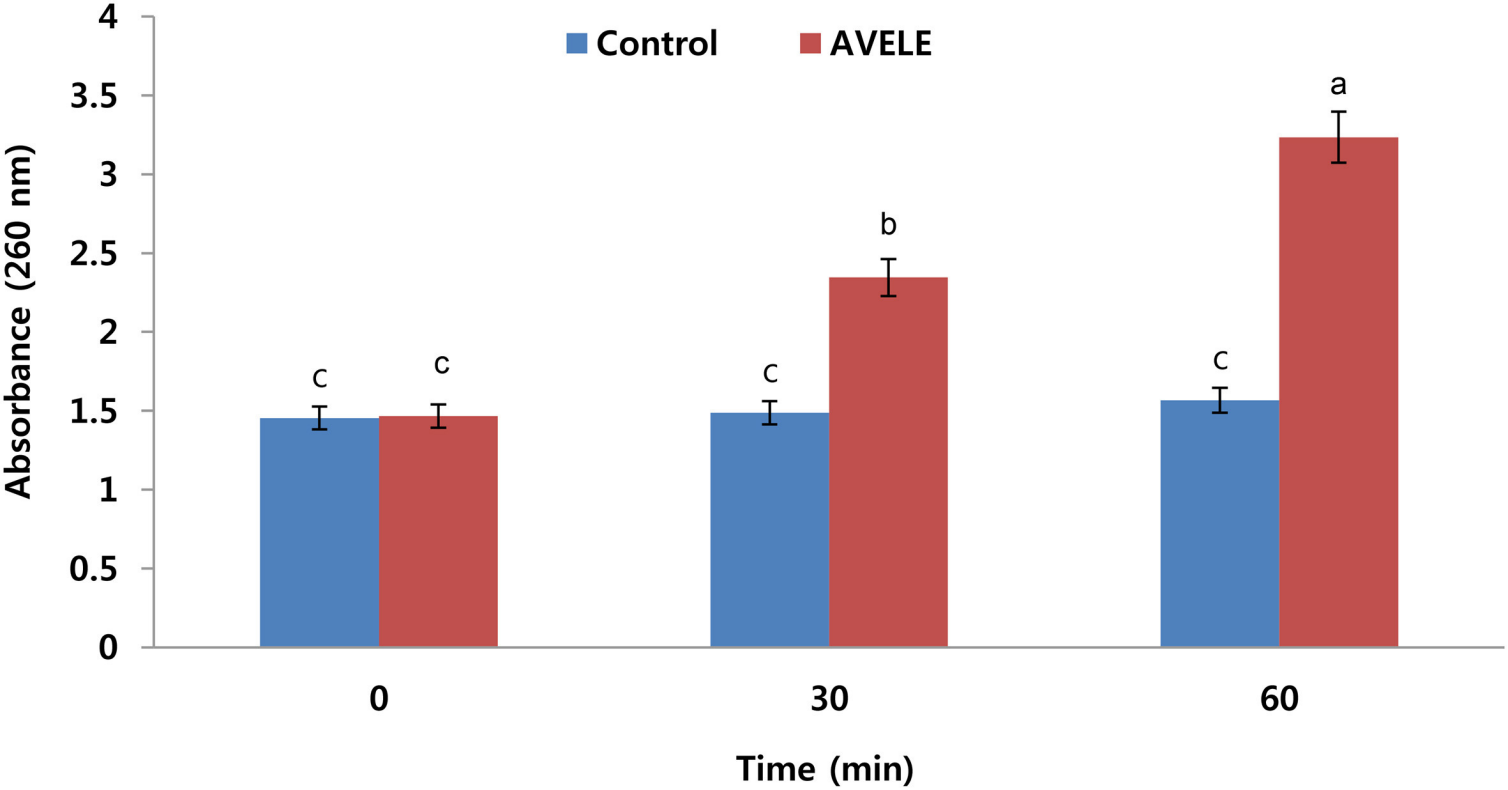
Effect of *Adhatoda vasica* ethanolic leaf extract (AVELE) on the release rate of 260-nm absorbing materials from the tested foodborne pathogen, *L. monocytogenes* NCIM 24563. The data are expressed as the mean ± *SD* (*n* = 3). The values in the same column with different superscripts are significantly different according to a Duncan's multiple range test (*p* < 0.05).

The investigation on the antibacterial effect of AVELE was extended to determine the nature of its possible anti-listerial mechanism. Although the synergistic mechanisms underlying the activities of various plant extracts are not well-understood, it was assumed that membrane perturbation and the adverse oxido-reduction potential by bioactive components, such as flavonoids, phenolics, and alkaloids, play major roles in establishing their antimicrobial effects (Walden and Hentges, 1975). Figure 3 shows the effects of AVELE at its MIC on the release of 260-nm absorbing materials from *L. monocytogenes* NCIM 24563. *L. monocytogenes* cells treated with AVELE displayed a time-dependent increase in absorbance, whereas the control samples showed no remarkable change (Figures 2, 3).

As a damaged bacterial cell membrane is more permeable, small molecules, such as phosphate and potassium ions, leach out from the cells before larger molecules, such as DNA and RNA (Chen et al., 2002), which absorb strongly at 260 nm, and are often referred to as “260-nm absorbing materials.” As genetic nucleotide materials, including DNA substances absorb maximum light at 260 nm, therefore, considering this fact, here we are measuring released DNA material from bacterial cell after treatment of AVELE extract. Furthermore, the release of these materials from bacterial cells is an indicator of a lack of membrane integrity (Bajpai et al., 2014). In the present UV-VIS study, the release of 260 nm-absorbing materials was proportional to the AVELE concentration, whereas the control cells showed no release of these materials after 120 min of exposure. These results show that AVELE induced the rapid losses of 260-nm-absorbing materials from *L. monocytogenes* NCIM 24563, indicating irreversible damage to the cytoplasmic membrane.

### Effect of AVELE on ATP concentration

**Figure 4:**
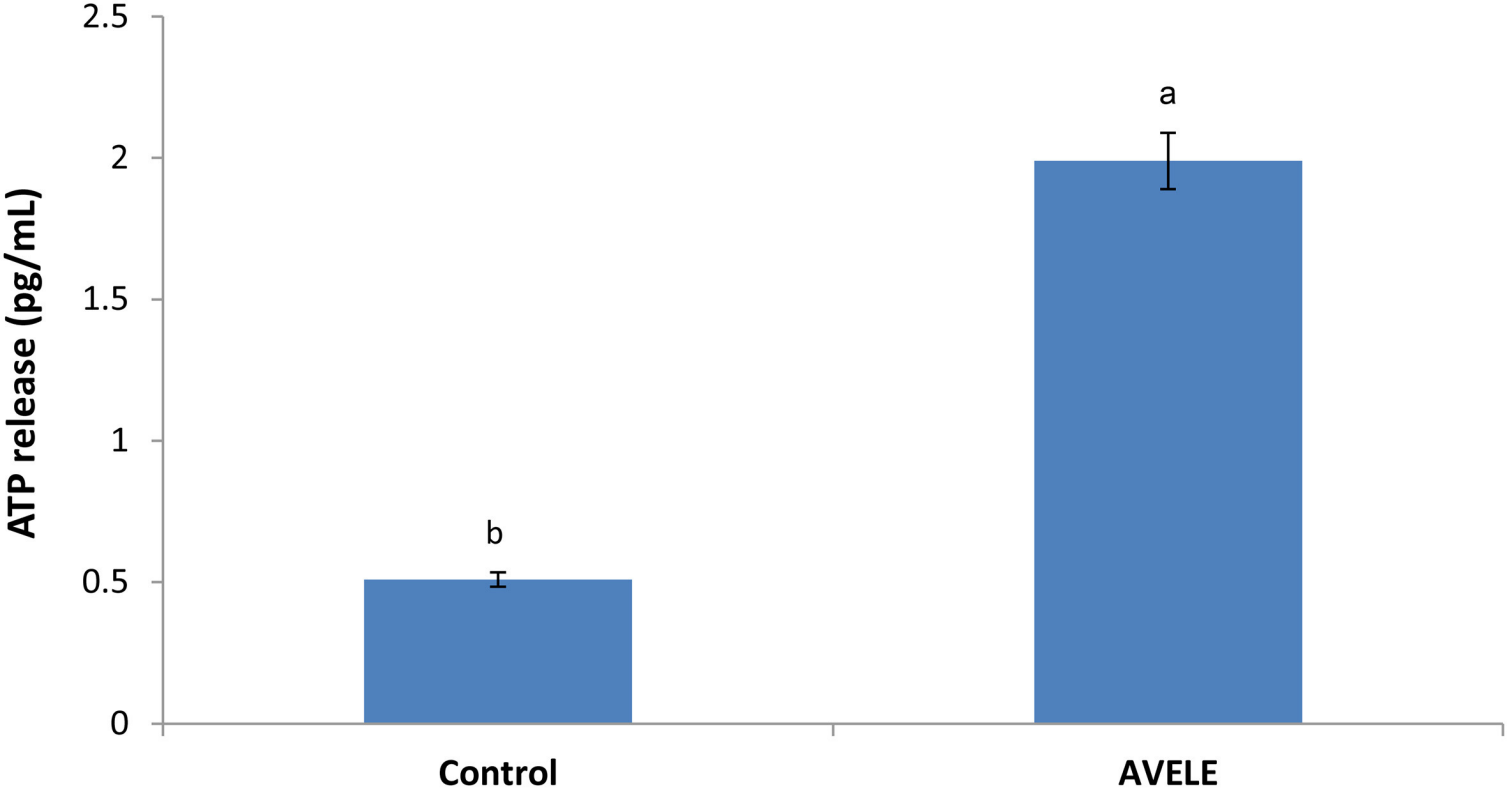
Effect of *Adhatoda vasica* ethanolic leaf extract (AVELE) on the release of extracellular ATP concentration from the cells of *L. monocytogenes* NCIM 24563. Data expressed as mean ± *SD* (*n* = 3). Values in the same column with different superscripts are significantly different according to Duncan's multiple range test (*p* < 0.05).

Figure 4 shows the effects of the MIC of AVELE on the extracellular ATP concentration in the cells of *L. monocytogenes* NCIM 24563. The concentrations of extracellular ATP in the control and treated cells of *L. monocytogenes* NCIM 24563 were 0.51 and 1.99 pg/mL, respectively. In this assay, AVELE showed a significant (*p* < 0.05) increase in the release of extracellular ATP from the target bacterial cells at the MIC when to the control group.

Previous studies have confirmed the efficacy of specific antimicrobial agents on the release of extracellular ATP loss from the target cells of foodborne pathogens, showing that antimicrobials can disrupt the bacterial cell membrane, thereby causing the loss of extracellular ATP from the target cells (Abee et al., 1994). As ATP contributes significantly to a number of cell functions, including the transportation of essential ions and metabolites across the cell-membrane, the excessive loss of ATP through a disruption of the membrane permeability might be a positive indication of the loss of cell membrane integrity, thereby causing cell death, as reported previously (Herranz et al., 2001; Bajpai et al., 2014).

### Application of AVELE as a rinse for chicken meat preservation and microbial analysis

All weighed samples of chicken meat were enriched and plated to confirm the presence or absence of *L. monocytogenes* NCIM 24563 prior to artificial inoculation; no prior contamination was detected. AVELE at concentrations of 50, 100, and 200 mg/mL was examined for its anti-listerial effect as a rinse on the raw chicken breast meat samples. *L. monocytogenes* counts were reduced significantly when the meat samples were rinsed with AVELE at 50 mg/mL, but they were not eliminated completely (Table 3). On the other hand, AVELE at 200 mg/mL for 90 min eradicated *L. monocytogenes* NCIM 24563 completely. Chao and Yin (2009) also observed that a *Hibiscus* extract at 10 mg/mL had a far greater antimicrobial effect against foodborne pathogens than at 5 mg/mL. In the current study, AVELE at concentrations of 50 and 100 mg/ml for 24 h could reduce the viable cell counts of *L. monocytogenes* (Table 3A). Higginbotham et al. (2014) also reported the anti-listerial effects of a *Hibiscus* rinse solution at 120 and 240 mg/mL on artificially contaminated hot dogs and found that both rinse solutions could reduce and/or eradicate the bacterial counts after a rinse treatment of 60 min and a storage time of 24 h. Based on the above findings, the mechanism of the reduction of the *L. monocytogenes* NCIM 24563 cell count using AVELE was time-dependent; a longer treatment time of AVELE reduced the cell numbers significantly (Tables 3A,B). Another study reported the inhibitory effects of dry seasoning treatment of chicken meat products on the survival of bacterial foodborne pathogens (Perko-Mäkelä et al., 2000). Several other researchers determined the inhibitory effects of various semisolid and liquid marinades added to chicken filets and observed a significant cell count reduction of a foodborne pathogen, *Campylobacter jejuni* (Birk et al., 2010).

**Table 3A:** Log CFU/g of *Listeria monocytogenes* NCIM 24563 and *Salmonella typhimurium* ATCC 43174 plated on agar plate following a rinse (5, 10, 15, 30, 60, and 90 min) with either distilled water or *Adhatoda vasica* ethanolic leaf extract (AVELE) (50, 100, and 200 mg/mL) in artificially inoculated raw chicken breast meat after 24 h storage at 4°C.

**Bacterial strains**	**Rinse time (min)**	**Control (water)**	**5% AVELE (50 mg/mL)**	**10% AVELE (100 mg/mL)**	**20% AVELE (200 mg/mL)**
*Listeria monocytogenes* NCIM 24563	5	6.5 ± 0.30^a^	6.5 ± 0.01^a^	6.1 ± 0.34^a^	6.0 ± 0.28^a^
	10	6.52 ± 0.25^a^	6.71 ± 0.21^b^	6.6 ± 0.02^b^	6.0 ± 0.3^a^
	15	6.5 ± 0.01^a^	6.8 ± 0.01^b^	6.2 ± 0.03^c^	5.1 ± 0.32^b^
	30	6.55 ± 0.02^a^	4.8 ± 0.04^c^	4.5 ± 0.12^d^	3.7 ± 0.10^c^
	60	6.6 ± 0.30^b^	4.2 ± 0.3^d^	3.6 ± 0.06^e^	2.2 ± 0.21^d^
	90	6.6 ± 0.21^b^	3.3 ± 0.22^e^	2.0 ± 0.12^f^	ND
*Salmonella typhimurium* ATCC 43174	5	6.8 ± 0.14^a^	6.5 ± 0.11^a^	6.5 ± 0.02^a^	6.5 ± 0.21^a^
	10	6.7 ± 0.03^a^	6.38 ± 0.32^a^	6.36 ± 0.02^a^	6.44 ± 0.16^a^
	15	6.6 ± 0.01^b^	6.0 ± 0.06^b^	5.6 ± 0.13^b^	5.1 ± 0.08^b^
	30	6.6 ± 0.21^a^	5.7 ± 0.43^c^	5.41 ± 0.06^b^	5.0 ± 0.02^b^
	60	6.6 ± 0.03^a^	5.5 ± 0.11^c^	5.32 ± 0.02^b^	4.4 ± 0.17^c^
	90	6.57 ± 0.11^b^	5.4 ± 0.18^c^	5.30 ± 0.01^b^	4.0 ± 0.12^d^

*ND, Colonies not detected. Values in the same column with different superscripts are significantly different according to Duncan's multiple range test (p < 0.05)*.

**Table 3B:** Log CFU/g of *L. monocytogenes* NCIM 24563 and *S. typhimurium* ATCC 43174 plated on an agar plate following a rinse (5, 10, 15, 30, 60, and 90 min) with either water or *A. vasica* ethanolic leaf extract (AVELE) (50, 100, and 200 mg/mL) in artificially inoculated pork meat and ready-to eat-salad after 24 h storage at 4°C.

**Bacterial strains**	**Rinse time (min)**	**Control (water)**	**5% AVELE (50 mg/mL)**	**10% AVELE (100 mg/mL)**	**20% AVELE (200 mg/mL)**
**ARTIFICIALLY INOCULATED PORK MEAT**					
*Listeria monocytogenes* NCIM 24563	5	6.0 ± 0.01^a^	6.2 ± 0.01^a^	6.2 ± 0.34^a^	6.0 ± 0.11^a^
	10	6.2 ± 0.22^a^	6.6 ± 0.31^b^	6.4 ± 0.42^b^	5.0 ± 0.23^b^
	15	6.2 ± 0.11^a^	6.6 ± 0.00^b^	6.7 ± 0.13^c^	4.4 ± 0.43^c^
	30	6.57 ± 0.32^b^	5.6 ± 0.16^c^	4.0 ± 0.22^d^	3.5 ± 0.00^d^
	60	6.6 ± 0.10^b^	4.5 ± 0.3^d^	3.2 ± 0.43^e^	2.8 ± 0.01^e^
	90	6.5 ± 0.22^c^	4.0 ± 0.12^e^	1.6 ± 0.42^f^	ND
*Salmonella typhimurium* ATCC 43174	5	6.8 ± 0.14^a^	6.5 ± 0.10^a^	6.5 ± 0.02^a^	6.5 ± 0.21^a^
	10	6.7 ± 0.43^a^	6.68 ± 0.12^a^	6.36 ± 0.02^a^	6.44 ± 0.16^a^
	15	6.6 ± 0.21^b^	5.8 ± 0.16^b^	5.6 ± 0.13^b^	5.1 ± 0.08^b^
	30	6.6 ± 0.21^b^	5.7 ± 0.22^c^	5.41 ± 0.06^b^	5.0 ± 0.02^b^
	60	6.5 ± 0.23^b^	5.6 ± 0.21^c^	5.32 ± 0.02^b^	5.0 ± 0.17^b^
	90	6.63 ± 0.41^b^	5.0 ± 0.12^d^	5.30 ± 0.01^b^	4.6 ± 0.12^c^
**READY-TO-EAT FRESH CUT SALAD**					
*Listeria monocytogenes* NCIM 24563	5	6.0 ± 0.01^a^	6.0 ± 0.11^a^	6.2 ± 0.20^a^	6.0 ± 0.51^a^
	10	6.2 ± 0.22^a^	6.2 ± 0.21^a^	6.2 ± 0.11^a^	6.0 ± 0.63^a^
	15	6.2 ± 0.11^a^	6.21 ± 0.03^a^	6.5 ± 0.64^b^	6.2 ± 0.44^a^
	30	6.57 ± 0.32^b^	6.0 ± 0.38^b^	6.0 ± 0.51^c^	6.0 ± 0.71^a^
	60	6.6 ± 0.10^b^	5.5 ± 0.04^c^	5.8 ± 0.54^c^	5.5 ± 0.21^b^
	90	6.5 ± 0.22^c^	5.5 ± 0.03^c^	5.5 ± 0.63^d^	5.3 ± 0.04^b^
*Salmonella typhimurium* ATCC 43174	5	6.8 ± 0.14^a^	6.3 ± 0.10^a^	6.6 ± 0.11^a^	6.2 ± 0.33^a^
	10	6.7 ± 0.43^a^	6.4 ± 0.12^a^	6.6 ± 0.48^a^	6.0 ± 0.06^a^
	15	6.6 ± 0.21^b^	6.8 ± 0.16^b^	6.0 ± 0.06^b^	5.6 ± 0.18^b^
	30	6.6 ± 0.21^b^	6.5 ± 0.22^c^	5.71 ± 0.14^c^	5.5 ± 0.64^b^
	60	6.5 ± 0.23^b^	6.3 ± 0.21^c^	5.6 ± 0.23^c^	5.2 ± 0.45^c^
	90	6.63 ± 0.41^b^	6.0 ± 0.12^d^	5.6 ± 0.11^c^	5.1 ± 0.07^c^

*ND, Colonies not detected. Values in the same column with different superscripts are significantly different according to Duncan's multiple range test (p < 0.05)*.

Two important aspects of the application of AVELE as a rinse are its antimicrobial effect on the meat sample and the acceptance of the treatment by the consumers. The use of naturally occurring antimicrobial agents in food system with enhanced functional properties has attracted considerable attention because these might be easily accepted by consumers. A better understanding of how bacteria cope with stress (e.g., resistance to antimicrobial compounds with different target sites in the cell, such as plant phenolic extracts), and adapt to a protective environment (e.g., chicken meat components), will be critical in the design of new (combined) intervention strategies and control methods for food safety management.

Various other rinses, such as acetylpyridinium chloride, have also been examined for their ability to inactivate harmful pathogens on ready-to-eat meat products. For example, spraying cetylpyridinium chloride at a concentration of 1% could reduce the *L. monocytogenes* NCIM 24563 counts on frankfurters (Singh et al., 2005). On the other hand, cetylpyridinium chloride is a synthetic compound, and despite its equivalent efficacy to those of plant extracts, such as *Hibiscus* extracts, it does not have the same appeal as natural products (Higginbotham et al., 2014). Similarly, Byelashov et al. (2008) reported that a combination of 5% lactic acid and 0.5% sodium lauryl sulfate applied to artificially contaminated frankfurters resulted in a significant decrease in the viable counts of *L. monocytogenes* NCIM 24563 after storage for 90 days.

In addition, in the present study, cell growth was analyzed for longer periods (36 and 48 h). As a result, there were no significant differences in the cell counts of *L. monocytogenes* NCIM 24563 and *S. typhimurium* ATCC 43174 contaminated food matrices after 24, 36, and 48 h. Changes in cell counts were noted after treatment with different AVELE rinsing solutions for different exposure times, which confirms that contact with the antimicrobial rinse solution affected the reduction in cell counts of *L. monocytogenes* NCIM 24563 on meat samples without any further regrowth in the bacterial community. Overall, a higher extract concentration, longer rinse time, with longer storage period (24, 36, and 48 h) were the most effective treatments for inhibiting and/or killing *L. monocytogenes* NCIM 24563 on chicken meat. These findings suggest that the use of an AVELE solution at an appropriate concentration (100 mg/mL) as a rinse agent might provide a feasible means of controlling *L. monocytogenes* NCIM 24563 contamination without adversely affecting the sensory quality of the tested meat products.

### Sensory analysis of chicken meat samples

The AVELE rinse-treated and non-treated chicken samples were cooked. After cooking, the samples were then cooled to room temperature for 1 h and analyzed for their color, and sensory characteristics. Table 4 lists the effects of the rinsing time and rinse concentration on the sensory properties of treated and non-treated, cooked chicken samples. At higher concentrations (200 mg/mL), although AVELE showed complete killing of the bacterial population of *L. monocytogenes* NCIM 24563, the flavor and taste of the resulting cooked chicken meat gave poor sensory scores. The consumers reported that it had a bitter taste with unacceptable color attributes (Table 4). Zhang et al. (2017) also reported that natural preservatives in larger amounts may alter the original food flavor in some cases, which is unacceptable to some consumers. Overall, the sensory properties were affected by the rinsing time and concentration, with almost all sensory attributes affected, including the meat tenderness, juiciness, taste, and flavor. The pH of the rinse solution was also reported to be 5.6–6.2, which does not have any effect on the adverse sensory and color attributes (data not shown). Based on the sensory analysis results, 5 and 10% AVELE (50 and 100 mg/mL) for a longer rinsing time (90 min) did not have adverse effects on the sensory attributes compared to untreated chicken samples and showed better tenderness with an overall consumer acceptability score (7.4–8.3) of the rinse-treated chicken samples than the other treatment groups.

**Table 4:** Sensory evaluation and Hunter's color value of an AVELE rinse solution treated, cooked chicken meat for overall consumers acceptability point view.

**Rinse type**	**Rinse time**	**Taste**	**Odor**	**Flavor**	**Tenderness**	**Color**	**Overall acceptability**	**Hunter's color values**		
								L^*^	a^*^	b^*^
Control	–	7.2 ± 0.14	8.4 ± 0.05	8.1 ± 1.22	5.0 ± 1.1	7.8 ± 1.21	8.2 ± 0.41	67.6 ± 1.43	25.4 ± 3.76	13.8 ± 6.65
R_1_(50 mg/mL)	5	7.0 ± 0.34	8.2 ± 1.2	7.8 ± 0.18	5.0 ± 1.22	7.0 ± 0.81	7.4 ± 0.08	67.2 ± 5.78	24.4 ± 7.22	15.1 ± 7.55
	10	6.6 ± 0.14	7.4 ± 0.76	7.4 ± 0.21	5.5 ± 0.23	7.4 ± 0.16	7.0 ± 0.13	65.6 ± 4.09	25.0 ± 4.81	15.5 ± 7.59
	15	6.7 ± 0.4	8.0 ± 0.88	7.3 ± 0.11	5.8 ± 0.11	7.7 ± 0.72	7.3 ± 0.22	64.0 ± 4.18	25.7 ± 5.13	15.8 ± 7.98
	30	6.6 ± 0.33	7.8 ± 0.24	7.5 ± 0.12	6.2 ± 0.31	7.8 ± 0.54	7.6 ± 0.12	64.5 ± 7.12	26.8 ± 5.14	15.7 ± 6.00
	60	6.8 ± 0.10	8.4 ± 0.31	7.8 ± 1.18	6.0 ± 0.83	7.8 ± 0.21	8.0 ± 0.10	63.0 ± 4.12	27.3 ± 5.03	15.6 ± 6.90
	90	6.7 ± 0.40	8.2 ± 0.33	7.4 ± 1.6	6.4 ± 0.44	7.8 ± 0.11	7.9 ± 0.18	62.7 ± 3.56	27.8 ± 6.19	15.8 ± 6.44
R_2_(100 mg/mL)	5	6.6 ± 0.21	7.8 ± 0.61	7.8 ± 0.03	6.3 ± 0.32	7.0 ± 0.52	7.7 ± 0.16	62.5 ± 7.22	25.4 ± 4.99	14.2 ± 4.86
	10	6.5 ± 0.17	7.7 ± 0.55	7.8 ± 0.13	8.0 ± 0.36	7.0 ± 0.61	8.0 ± 0.18	62.9 ± 5.84	26.3 ± 7.26	14.8 ± 4.85
	15	6.8 ± 1.10	8.0 ± 0.12	7.7 ± 0.32	8.2 ± 0.04	7.5 ± 0.13	8.0 ± 0.21	63.0 ± 5.87	27.0 ± 4.96	15.4 ± 4.74
	30	6.7 ± 0.02	8.0 ± 0.31	8.0 ± 0.41	8.2 ± 0.31	7.7 ± 0.17	8.2 ± 0.18	63.3 ± 8.34	27.7 ± 4.61	15.8 ± 5.56
	60	7.0 ± 1.1	8.6 ± 0.11	8.0 ± 0.55	8.3 ± 0.51	8.0 ± 0.10	8.3 ± 0.20	63.1 ± 2.21	28.5 ± 5.18	15.2 ± 3.45
	90	6.8 ± 0.8	7.0 ± 0.12	7.4 ± 1.14	8.4 ± 0.63	7.6 ± 1.10	7.0 ± 1.16	61.3 ± 6.43	26.1 ± 4.96	15.0 ± 3.75
R_3_(100 mg/mL)	5	5.1 ± 0.45	5.2 ± 0.41	5.0 ± 0.17	7.8 ± 0.21	7.2 ± 0.21	6.4 ± 0.81	61.0 ± 6.98	26.1 ± 4.89	15.4 ± 5.15
	10	5.0 ± 0.34	5.0 ± 0.18	4.5 ± 0.35	6.6 ± 0.33	5.6 ± 0.41	5.7 ± 0.92	56.5 ± 4.67	27.3 ± 4.97	15.9 ± 4.60
	15	5.0 ± 0.11	5.2 ± 0.19	4.8 ± 0.41	6.6 ± 0.12	5.7 ± 0.52	5.0 ± 0.23	53.2 ± 5.06	27.7 ± 6.55	16.3 ± 4.65
	30	5.8 ± 0.31	4.4 ± 0.20	4.5 ± 1.14	7.4 ± 0.22	5.0 ± 0.61	5.5 ± 0.31	51.7 ± 4.44	28.1 ± 7.21	16.6 ± 5.11
	60	5.0 ± 0.42	4.2 ± 0.16	4.7 ± 0.43	7.4 ± 0.18	5.6 ± 1.82	5.2 ± 0.62	50.3 ± 4.81	28.6 ± 6.32	16.8 ± 5.13
	90	5.5 ± 0.13	3.6 ± 0.21	3.9 ± 1.13	7.8 ± 0.81	4.5 ± 1.18	5.0 ± 0.13	48.5 ± 4.66	28.9 ± 6.98	17.4 ± 4.96

*The experiments were performed in triplicate and values are presented as the mean ± SD*.

### Color quality of cooked chicken

The color quality of the AVELE rinse-treated chicken meat samples differed slightly in lightness (L^*^), redness (a^*^), and yellowness (b^*^) from the control (untreated chicken samples). The rinsing concentration and rinse time were closely related to the color change in the cooked chicken samples. The 5% and 10% AVELE (50 and 100 mg/mL) for a rinsing time of 60 and 90 min showed a significantly low L^*^ (lightness) value, and high a^*^ (redness), and yellowness values (b^*^) compared to the untreated and the AVELE treatments for a 30 min rinsing time (*p* < 0.05) (Table 4), suggesting a positive correlation between the L^*^ (lightness) and a^*^ (redness) values upon the increasing chicken rinsing time. During sensory analysis, the color of the AVELE treated, cooked chicken was also one of the attributes for its consumer acceptability. In the present study, a higher concentration of AVELE rinse solution (20%) showed significant differences in color values than the other concentrations of AVELE and non-treated chicken samples, which could not meet the consumer acceptability criteria due to the appearance of chicken meat samples during the sensory evaluation. Yusop et al. (2010) also reported that prolonged marinating or rinsing times using Chinese-style marinades and rinse solutions had significant effects in producing a product with lower L^*^ (lightness) and higher a^*^ (redness) values.

### Effects of AVELE rinse solution on other food matrices contaminated with *L. monocytogenes* NCIM 24563 and *S. typhimurium* ATCC 43174

In this study, AVELE was more active in eradicating *L. monocytogenes* NCIM 24563 than *S. typhimurium* ATCC 43174 when the chicken samples were treated with the AVELE rinse solutions (50, 100, and 200 mg/mL). Surprisingly, in preliminary studies, AVELE could reduce the *S. typhimurium* ATCC 43174 counts in pure cultures but during its applicability as a rinse on chicken meat, it did not show a significant reduction in the cell counts of *S. typhimurium* ATCC 43174 except for a very minor cell count reduction, suggesting that there might be some interference due to the components of chicken meat food matrices, such as proteins or fats (Table 3A). Similarly, Friedman et al. (2017) reported that plant extracts rich in phytochemicals had inhibitory potential against *E. coli* O157:H7 but not against *Salmonella enterica* in an essential oil/wine mixture, suggesting some synergistic effects of the phytochemicals present in plant extracts, components of added essential oils or natively present in the food wine (Rota et al., 2008).

In addition, the colony counts, even after the addition of AVELE to the meat samples, showed a similar or higher level of contamination over a few minutes, which were reduced gradually (Tables 3A,B). This is called the phoenix effect, where a few microbial cells survive for a long lag phase and then begin to regrow. A similar reduction rate in the microbial counts has been observed in many food products (Subramaniam et al., 2015).

This study tested the efficacy of AVELE as a rinse with few other food matrices, including pork meat and ready-to-eat fresh cut salad against *L. monocytogenes* NCIM 24563 and *S. typhimurium* ATCC 43174. A significant decrease in the cell counts of *L. monocytogenes* NCIM 24563 and *S. typhimurium* ATCC 43174 compared to the control (without treatment) was observed. A comparison of the rinsing effect of AVELE with different food matrices revealed a strong reduction in the cell counts in the case of only chicken and pork meat samples contaminated with NCIM 24563 than *S. typhimurium* ATCC 43174 in all food matrices (Table 3B). AVELE, as a rinse, had no reducing effect on the bacterial counts of *L. monocytogenes* NCIM 24563 and *Salmonella* ATCC 43174 in ready-to-eat fresh cut salad. This might have occurred due to the variations in the composition of the different food matrices, such as chicken and pork meat, which are similarly rich in protein and fat, compared to the ready-to-eat fresh cut salad. This highlights the applicability of AVELE as a rinse in a variety of food matrices contaminated with different foodborne pathogenic microflora, particularly *L. monocytogenes*.

## Conclusion

The ethanolic leaf extract of *A. vasica* (AVELE) showed significant antibacterial activity, as evidenced by its inhibitory effects on bacterial growth, cell viability, potassium ion efflux, release of 260-nm absorbing materials, and loss of the extracellular ATP pool. Moreover, a AVELE solution as a rinse/marinade effectively decreased the bacterial counts of *L. monocytogenes* inoculated into raw and processed minced chicken meat samples; this was attributed tentatively to its phenolic, flavonoid, and alkaloid contents. Furthermore, AVELE, as a rinse, could enhance the nutritional value of meat products, as confirmed by the sensory scores with improved tenderness, juiciness, and flavor of the meat samples. These findings reinforce the suggestions that AVELE improved the safety and quality of ready-to-eat and raw meat products by inhibiting *L. monocytogenes*, and can be recommended as a rinse or marinade for future applications in the food industry with enhanced consumer acceptability.

## Author contributions

SS, LA, and VB performed experiments and wrote the manuscript; YSH and YKH: designed the experimental strategy, and critically reviewed the manuscript.

### Conflict of interest statement

The authors declare that the research was conducted in the absence of any commercial or financial relationships that could be construed as a potential conflict of interest.
