# Corrigendum: Growth Inhibitory Effects of *Adhatoda vasica* and Its Potential at Reducing *Listeria monocytogenes* in Chicken Meat

**DOI:** 10.3389/fmicb.2017.01636

**Published:** 2017-08-24

**Authors:** Shruti Shukla, Laxmi Ahirwal, Vivek K. Bajpai, Yun Suk Huh, Young-Kyu Han

**Affiliations:** ^1^Department of Energy and Materials Engineering, Dongguk University Seoul Seoul, South Korea; ^2^Laboratory of Plant Pathology and Microbiology, Department of Botany, Dr. Hari Singh Gour University Sagar, India; ^3^Department of Applied Microbiology and Biotechnology, Yeungnam University Gyeongsan-si, South Korea; ^4^Department of Biological Engineering, Inha University Incheon, South Korea

**Keywords:** *L. monocytogenes* NCIM 24563, *Adhatoda vasica*, ethanolic extract, antimicrobial, food model

In the original article, we neglected to include the funder the Basic Science Research Program through the National Research Foundation of Korea (NRF) funded by the Ministry of Science, ICT & Future Planning, 2017R1D1A1B03035373 to Shruti Shukla. The authors apologize for this error and state that this does not change the scientific conclusions of the article in any way.

## Conflict of interest statement

The authors declare that the research was conducted in the absence of any commercial or financial relationships that could be construed as a potential conflict of interest.

